# Effects of Playing a Serious Computer Game on Body Mass Index and Nutrition Knowledge in Women

**DOI:** 10.2196/games.4977

**Published:** 2016-06-02

**Authors:** Mariya Shiyko, Sean Hallinan, Magy Seif El-Nasr, Shree Subramanian, Carmen Castaneda-Sceppa

**Affiliations:** ^1^ Northeastern University Applied Psychology Boston, MA United States; ^2^ Northeastern University College of Computer and Information Science Boston, MA United States; ^3^ Northeastern University Center for Advancing Teaching and Learning through Research Boston, MA United States; ^4^ Northeastern University Department of Health Sciences Boston, MA United States

**Keywords:** serious games, games for health, weight loss, body mass index, nutritional knowledge, game play, self-determination theory, Player Experience of Need Satisfaction (PENS) model, women

## Abstract

**Background:**

Obesity and weight gain is a critical public health concern. Serious digital games are gaining popularity in the context of health interventions. They use persuasive and fun design features to engage users in health-related behaviors in a non-game context. As a young field, research about effectiveness and acceptability of such games for weight loss is sparse.

**Objective:**

The goal of this study was to evaluate real-world play patterns of SpaPlay and its impact on body mass index (BMI) and nutritional knowledge. SpaPlay is a computer game designed to help women adopt healthier dietary and exercise behaviors, developed based on Self-Determination theory and the Player Experience of Need Satisfaction (PENS) model. Progress in the game is tied to real-life activities (e.g., eating a healthy snack, taking a flight of stairs).

**Methods:**

We recruited 47 women to partake in a within-subject 90-day longitudinal study, with assessments taken at baseline, 1-, 2-, and 3- months. Women were on average, 29.8 years old (±7.3), highly educated (80.9% had BA or higher), 39% non-White, baseline BMI 26.98 (±5.6), who reported at least contemplating making changes in their diet and exercise routine based on the Stages of Change Model. We computed 9 indices from game utilization data to evaluate game play. We used general linear models to examine inter-individual differences between levels of play, and multilevel models to assess temporal changes in BMI and nutritional knowledge.

**Results:**

Patterns of game play were mixed. Participants who reported being in the preparation or action stages of behavior change exhibited more days of play and more play regularity compared to those who were in the contemplation stage. Additionally, women who reported playing video games 1-2 hours per session demonstrated more sparse game play. Brief activities, such as one-time actions related to physical activity or healthy food, were preferred over activities that require a longer commitment (e.g., taking stairs every day for a week). BMI decreased significantly (*P*<.001) from baseline to 3-month follow-up, yielding a large effect size of 1.28. Nutritional knowledge increased significantly (*P*<.001) from first to third month follow-ups, with an effect size of .86. The degree of change in both outcomes was related to game play, baseline readiness to change, and the extent of video game play in general.

**Conclusions:**

This work demonstrates initial evidence of success for using a serious game as an intervention for health behavior change in real world settings. Our findings also highlight the need to understand not only game effectiveness but also inter-individual differences. Individualizing content and the intervention medium appears to be necessary for a more personalized and long-lasting impact.

## Introduction

Obesity and weight gain are critical public health concerns in the United States. More than one third of all US adults are overweight, a total of 78.6 million [1]. Obesity has been strongly linked to several preventable health conditions, including heart disease, stroke, type 2 diabetes, certain types of cancers, sleep apnea, and hypertension, among others [2]. An estimated 300,000 deaths a year are linked to obesity [3]; and almost 21% of the total healthcare budget (around $190.2 billion) is spent annually towards obesity-related illnesses [4].

Given the scope of the problem, the Internet and ubiquitous technology present a unique opportunity for behavior change intervention and reach. Serious games targeting health behavior change represent a new field of research. Video games are a growing medium in the United States and are becoming more popular than the motion picture industry. Zynga, a large game developer, claims 148 million unique users [5]. Video games are now appearing on computers, phones, toys, and even medical devices and kitchen appliances [6]. As an intervention tool, video games are appealing due to the adaptability and customizability of the user’s experience, and tend to be relatively low-cost [6]. Serious games are a genre of video games that employ playful design strategies to encourage users’ engagement in a non-game context. They are considered to be uniquely suited for increasing individuals’ motivation and, thus, have a potential to reach individuals to whom traditional modalities of behavior change may not be appealing or available [7].They can also be scaled up to reach a large audience. Based on a recent meta-analysis, online behavior change technologies (which include video games) are more successful than public health campaigns at initiating behavior change, reaching 10% of users, compared to public health’s 5% [8].

While promising, there is scarce empirical evidence of the efficacy of serious games for weight loss in adults, as only a few studies have evaluated games in the adult population. Moller et al. [9] assessed the acceptability and initial effectiveness of an online fantasy sports game on physical activity in two small pilot studies, spanning 13 (N=9) and 17 (N=10) weeks respectively. An overall positive effect was found on maintenance and increase in walking behavior in a convenience sample of adults. Another study [10] evaluated the effect of a 3-week role-play educational game with a sample of 40 undergraduates (80% women) and found an improvement in knowledge of healthy foods, an enhanced understanding of the perceived benefits of and barriers to healthy eating, and increased self-efficacy and intention to engage in healthy eating behaviors. Finally, in the context of diabetes management, adults (N=41) participating in a virtual island game aimed to promote knowledge of health-related self-management behaviors demonstrated a modest significant weight loss over the period of 6 months [11]. The field is gradually developing, and a description of several other games is available (eg, Dance Dance Revolution [12], an exercise bicycle linked to a computer game [13], and a pedometer linked to a fish avatar [14]). However, there is no empirical data to evaluate effectiveness of these games, although a few initial studies provide some evidence for the effectiveness of serious games as ways to increase weight loss-related health behaviors in adults.

Despite the initial promise of serious games, there is a need to understand their role, applications, limitations, and types of individuals for whom they are most suitable. Technology on its own is unlikely to make games effective unless strongly grounded in the principles and theories of health behavior change. Some games are developed in collaboration between industry and academia, with theories of health behavior change serving as a foundation for game design [15]; however, this trend is still in its infancy. Based on empirical evidence and theory [16-20], the following elements are considered important for building a persuasive product: goal setting, capacity to overcome challenges, providing feedback on performance, reinforcement, progress comparison, social connectivity, and fun and playfulness [21]. In games, the elements translate through gamification principles, defined as the use of game design elements in a non-game context [22]. Some examples of gamification include an engaging story line [23]; provision of clear goals and challenges through game principles of leveling up, earning points, badges, and rewards; a regular performance feedback through visualization; and community support through an in-game social network [21].

Weight loss and maintenance require regular engagement in healthy eating and physical activity over a long period. While some intervention programs have been shown to be effective in the short term [24], a relapse to old habits is common. For example, an estimated 94% relapse has been observed among people engaged in dieting [25]. Motivation to engage in health-related behaviors is essential and has been shown to be more effective long-term (ie, lasting 6-18 months) than a skills-based approach [26]. Self-determination theory is a global theory of human motivation that has been actively applied to video games [10,27-29]. The theory postulates that continuous motivation can be sustained through meeting 3 primary human needs: *competence*, *autonomy*, and *relatedness*. *Competence* is defined as the innate desire to learn new skills and gain mastery over them. It can be cultivated through presenting new challenges that progressively build learned skills, creating opportunities for participants to meet those challenges and progress through game leveling. *Autonomy* is defined as an innate desire to be in control over goals and behaviors. In a game, this can be achieved by allowing users to choose personal goals and individual behaviors that can meet the desired goals. Finally, *relatedness* taps into the human propensity for a social connection and belonging. Relatedness can be targeted through real or virtual social networks, players’ community support through feedback, encouragement, and competition.

Overall, self-determination theory is one of the frameworks that has been studied in the field of video games [30-31] and explicitly adopted as the Player Experience of Need Satisfaction (PENS) model, which outlines game elements that tap into needs for competence, autonomy, and relatedness. Based on research from thousands of players, video games that include elements that meet these needs are predictive of emotional, behavioral, and objective outcomes, including self-reported fun and enjoyment, game immersion, game values and sales, length of play, and recommendations to others [31]. To our knowledge, no existing games have explicitly incorporated elements of the PENS model into their design to reduce the Body Mass Index (BMI) and increase nutrition knowledge. Given that weight loss requires a long-term commitment, motivation towards a healthy weight is an important factor to initiate and sustain new behaviors. It is our hypothesis that explicitly designing a game based on the PENS principles will also translate into real-life behavioral outcomes.

In this paper, we present SpaPlay–a serious game for encouraging and sustaining healthy living in women [27]. The game SpaPlay was developed based on the outlined principles of the PENS model [31] and gamification strategies [21,31]. Our previous qualitative study demonstrated overall acceptability of the game [32-33]. The focus of the current study is to evaluate the extent of game play derived from objective gamification data and examine initial evidence of play effectiveness on BMI and nutritional knowledge. In the following sections, we provide an overview of the game, describe the within-subject longitudinal pilot study, and summarize major findings and conclusions.

### SpaPlay–Game Description

**Figure 1:**
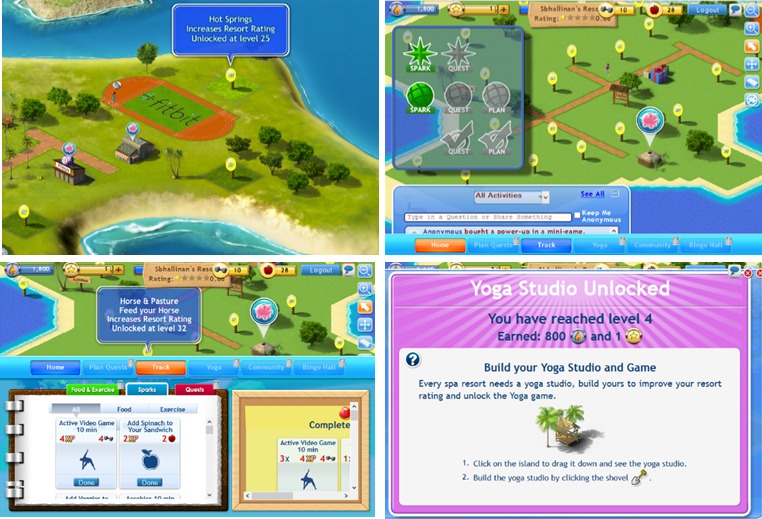
Screenshots of SpaPlay.

SpaPlay is a digital social online game developed to motivate women to make healthy eating choices and to exercise. It was developed through a close partnership between academia and industry, spanning more than 5 years, and involving all stages of game design and development. The current version of SpaPlay is a browser-based video game accessible to players via personal computers and laptops. It requires an Internet connection and a user-generated username and password. The content of the game centers on a virtual spa resort that needs to be developed and maintained, similar to other popular games like Farmville or We Rule. Figure 1 presents several screen shots of game elements. SpaPlay bridges real and virtual worlds, and game progress is contingent on activities completed in real life around physical activity and healthy eating. For example, eating a salad or taking a 10-minute walk would earn players points towards developing the spa (eg, building facilities, accumulating ratings, playing mini games). Gamification and principles of the Self-Determination theory are used to sustain players’ interest and engagement. Further, since weight loss takes time to self-observe, more immediate rewards in the game (eg, power-ups and customization options for the user’s avatar) or real life (eg, coupons from associated vendors) are used to keep players engaged.

**Figure 2:**
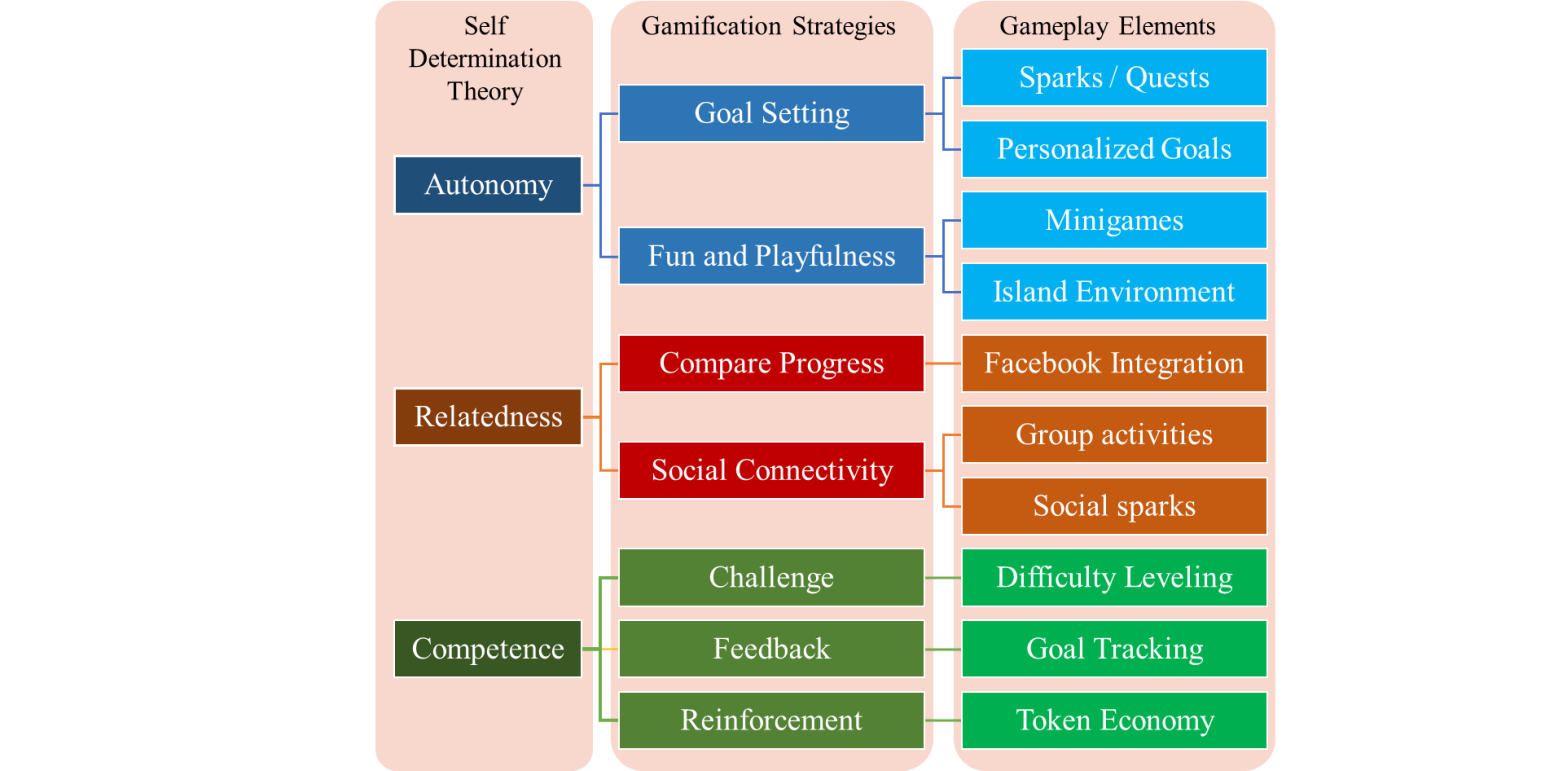
Self-Determination theory-based game elements in SpaPlay.

Figure 2 explicitly demonstrates the relationship between game play elements, gamification principles and the self-determination theory. The two core game mechanics through which experience points are earned are Quests and Sparks. Quests are sets of physical activities or dietary tasks that the players complete within the span of a week and target longer-term commitment to health behaviors. An example includes taking 1-2 flights of stairs twice within a week; substituting a sugary beverage for water 5 times a week; or eating a fruit instead of a snack twice in a week. Sparks are short, single-time tasks that can include stretching for 5 minutes or adding spinach to a sandwich. Social Sparks are dietary or exercise activities in which the user engages with others in the real world. Both Sparks and Quests allow users to set their own short and longer-term goals and chose from a variety of activities that the game offers. They promote autonomy in the way that the players design and enact their own program. They also build social connectivity through in-game and real-life group-based activities (eg, taking a walk with a friend), promoting relatedness.

It is important to note a few additional game features. For instance, healthy behaviors are introduced through the game interface, which both teaches exercise methods, such as yoga through the yoga mini game, and proper diet through recommendations and diet mini games, such as the chef game, which challenges players to create healthy dishes with healthy recipes. Self-tracking of physical activity is enabled through a connected Fitbit sensor, allowing users to log and measure external activities. Finally, the game incorporates social features that allow users to interact with friends and display measures of their progress.

## Methods

### Participants

To evaluate the game, we recruited women from an urban New England area. Participants self-selected for the study in response to online solicitations sent through listservs and handouts and flyers posted at several public health and non-for-profit organization sites. Inclusion criteria were being ≥ 21 years old, speaking English, and reporting being at least at the contemplation stage of change for engaging in exercise and nutrition on the transtheoretical model of change [34], as described below. Of the 60 women initially recruited, 13 (21.7%) dropped out within the first week of the study. Reasons cited included lack of gaming experience and inability to provide adequate time commitment to game play. The final sample was comprised of 47 women who partook in the 90-day longitudinal study, with global assessments taken at baseline, 1-, 2-, and 3- month follow-ups. The study was approved by the Institutional Review Board at Northeastern University.

### Procedure

After the online consent, participants completed a baseline survey and were given instructions with game access. Following the baseline, participants were contacted via emails at months 1, 2, and 3 with a request to fill out follow-up surveys assessing main study outcomes (BMI and nutrition knowledge). A research assistant emailed participants once a month to check on progress and address any questions or concerns. All participants were awarded a $20 gift certificate at study completion.

### Measures

Telemetry game play data were automatically recorded and time-stamped throughout the game play period. Data consisted of time-stamped actions, such as logins, type of Quests or Sparks selected, completion of Sparks or Quests, game activities such as picking up trash, score changes, and social interactions.

Two major study outcomes were BMI and nutritional knowledge. Participant self-reported weight and height was collected at baseline, 1-, 2-, and 3- months. BMI was calculated using the formula: weight (kg)/height (m)^2^ [35].

Nutritional knowledge was assessed at 1, 2, and 3 months with the General Nutrition Knowledge questionnaire for adults. The questionnaire is comprised of 53 items evaluating an individual’s knowledge of nutritional and dietary needs. It has high test-retest reliability and construct validity established through expert review [36]. To reduce burden, due to the length of the questionnaire, we purposefully omitted assessing participants on their nutrition knowledge at baseline.

Readiness to change behaviors in domains of exercise, nutrition and consumption of sweetened beverages was assessed at baseline by the *Readiness to Change Questionnaire*, a 16-item instrument with high levels of test-retest reliability and predictive validity for behavior change [37]. For this study, we omitted information on the beverage-related items, and readiness to change on exercise and nutrition domains were used as covariates.

### Statistical Analyses

**Figure 3:**
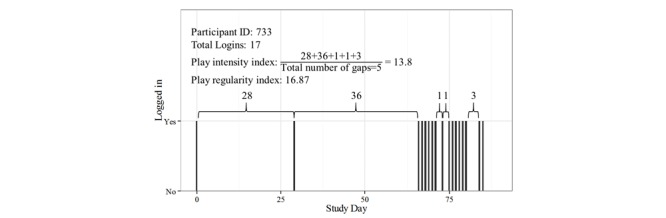
Example log-in data from one study participant. Vertical bars across the 90-day timeline represent occurrences of play, with quantitative summaries of adherence statistics.

First, we examined play patterns based on objective telemetry data. Frequency of play was determined from daily game logins, which were tracked from the automatically recorded telemetry data. Daily play was noted as present (1) or absent (0). The total number of play days was computed for each person. In addition, the *play intensity index* was computed as the length of time between logins. With daily play, an individual mean would be expected to equal 0. Less frequent play would naturally translate into a higher mean value. *Play regularity index* was computed as the standard deviation between logins. For a regular play pattern (eg, every 2 days), it would be expected to be 0, and increase with irregular play. Figure 3 presents an example of login data from one study participant with corresponding play intensity and play regularity indices. Further, to capture the nature of game activities for each individual, we computed the total number of food- and exercise- related Sparks and Quests, and the number of game and social activities. Descriptive statistics were computed for all 9 indicators of game play for the entire sample.

Second, we examined whether background baseline variables differentiated between play patterns. To account for data non-normality, log transformations were applied to total days of play and the total number of food and exercise Quests and Sparks. General linear models were run with 7 predictors including education, ethnicity, age, history of weight loss program participation, baseline BMI, history of gaming 1-2 hours per session, and baseline stage of change on exercise and healthy food intake questionnaires. While repeated testing jeopardizes the overall type I error rate, given the preliminary nature of the study, we did not adjust the p-value and kept it at the .05 level for each model.

**Figure 4:**
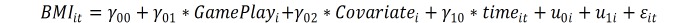
MLM equation.

Third, to examine changes in BMI and nutritional knowledge over the course of gameplay, multilevel modeling (MLM) [38,39] was used to test for effects of time and game adherence on the study outcomes while controlling for major demographics (ie, age, ethnicity, and educational level), history of game play, and baseline readiness to change stage. MLM is designed to account for nested data structure with observations clustered within participants, for missing data with some participants omitting certain observations, and for inter-individual differences in trajectories with possible variability in intercept and slope values. The following model, as specified in Figure 4 was fit to the data, where the residuals are normally distributed, ε_*it*_∼*N*(0,R_*i*_), with variance *R*_*i*_ following the compound symmetry structure, and *u*_*0i*_ and *u*_*1i*_ are random intercept and slope parameters with corresponding variances, *τ*_00_ and *τ*_10_. In this equation, BMI for a given person *i* measured at time *t* is modeled as a function of, where time is centered at the 3^rd^month follow-up (with values of -1, -2, and -3 corresponding to observations at 60 days, 30 days, and baseline, respectively). γ_00_ is the BMI value at 3 months, controlling for study covariates *Cov_i_*, γ_10_ is the effect of game play on BMI, and is a linear rate of change in BMI in increments of 30 days (1 month). A similar model was fitted to Nutrition Knowledge as the outcome. Both were fitted in the nonlinear mixed effects (nlme) package in R, which is free, open-source statistical software [40]. Final models included predictors significant at the .05 alpha level.

## Results

### Sample Description

The sample was comprised of women with an average age of 30 years (SD 7.3), 81% (38/47) reporting a Bachelors or Masters degree, 61% (30/47) being White, 20% (10/47) Asian, 8% (4/47) Black, and 2% (1/47) Latina. Of these participants, 6% (3/47) were concurrently enrolled in a weight loss program, while 32.7% (16/47) had previously completed weight loss programs. Average BMI at the beginning of the study was 26.98 (SD 5.6), which is considered “overweight” by the CDC’s criteria [35]. A number of participants (8/47, 17%) reported daily video game usage, 33% (26/47) reported less than daily but more than weekly usage, 25% (12/47) reported playing weekly or less frequently, and 6% (3/47) did not provide information about their video game behavior. Of those who did report playing video games, 72% (34/47) reported playing 1-2 hours per gaming session.

For behavior change stages on nutrition, 60% (28/47) of participants were in the contemplation stage, 34% (16/47) were in the preparation stage and 4% (247) were in the action stage. For physical activity, 55% (26/47) were in the contemplation stage, 38% (18/47) were in the preparation stage and 4% (2/47) were in the action stage.

### Game Play

Table 1 summarizes descriptive statistics for game play indicators. On average, participants played about 7 days (SD 12.5), 25% of participants did not utilize the game at all between the beginning and the end of the study, and 75% logged in fewer than 7 times over the course of the study. Food and exercise Sparks were the most popular activities. Distributions of Sparks were very skewed, with several players engaging in several hundreds of Sparks over the course of 90 days, but the majority engaging in a few (median of 8 for food and 4 for exercise Sparks). The average number of days between logins was 24.65 (SD 14.23), and the play regularity index averaged at 16.48 (SD 7.66).

**Table 1:** Summary of game play data.

Activity	Mean (SD)	Median
Total Number of Play Days	6.9 (12.5)	2
Play Intensity Index	24.65 (14.23)	22.04
Play Regularity Index	16.48 (7.66)	15.16
Food Sparks	108.2 (349.2)	4
Food Quests	4.5 (10.8)	0
Exercise Sparks	100.6 (294)	8
Exercise Quests	9.3 (27.5)	0
Game Activities	1.8 (3.2)	0
Social Activities	1.2 (2.4)	1

Analyses of game play data revealed several predictors that differentiated between different levels of engagement with the game (Table 2). Two major predictors emerged. First, those who reported being in the preparation or action stages of eating healthy at baseline had a higher total number of logins, had shorted gaps between logins, played the game more consistently, and completed more exercise Sparks (all *P* values < .05). Second, those who reported at baseline spending 1-2 hours playing video games per session had fewer total logins and completed fewer exercise and food-related Sparks and Quests (all *P* values < .05).

**Table 2:** Results of general linear models predicting game play data^a^.

Outcomes	Predictors: Parameter Estimate (Standard Error)			
	Intercept	Gaming 1-2 hr/session	TTM Eat Healthy	Below BA
Log (Days Total)	1.589 (.307)	-.943 (.386) *P*=.02	.789 (.321) *P*=.02	
Play Intensity	27.752 (2.497)		-8.953 (3.703) *P*=.02	
Play Regularity	18.403 (1.340)		-5.393 (1.988) *P*=.001	
Log (Food Sparks)	3.531 (1.214)	-3.067 (1.330) *P*=.046		-3.153 (1.535) *P*=.026
Log (Exercise Sparks)	2.573 (.947)	-2.769 (1.192) *P*=.025	2.667 (.991) *P*=.01	
Log (Food Quests)	-.446 (1.119)	-4.310 (1.312) *P*=.002		
Log (Exercise Quests)	-.234 (1.198)	-4.165 (1.404) *P*=.005		

* *P*<.05

** *P*<.01

^a^Results for Log (Game Activities) and Log (Social Activities) were not significant.

### Multilevel Modeling

**Figure 5:**
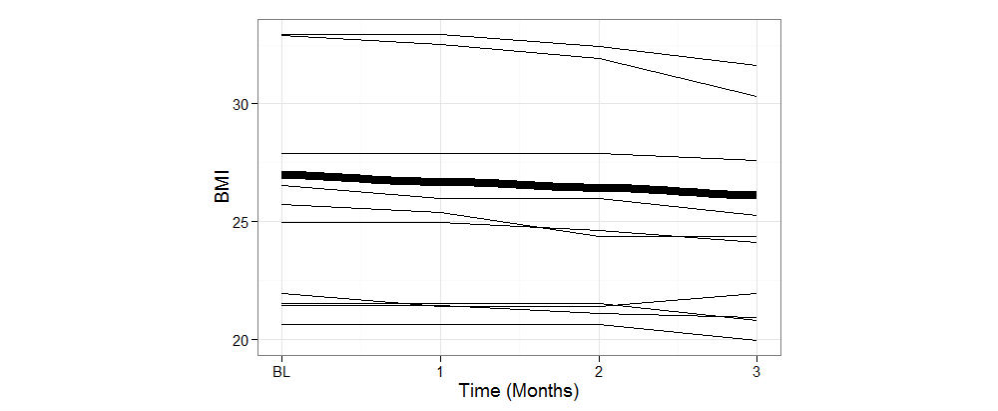
Graphical summary of BMI trajectories across the 3 study Months: Sample average (thick black line) and individual trajectories of ten randomly selected participants.

Figure 5 presents observed BMI trajectories, with the large line summarizing the sample average, and thinner lines summarizing data from 10 randomly selected participants. Overall, the trajectory decreased over the course of three months. Average BMI at the beginning of the study was 26.98 (SD 5.6) and 26.09 (SD 5.27) at the end of the study, corresponding to Cohen’s *d* effect size of 1.28 (large) based on a paired-sample *t* test.

Table 3 presents MLM results for BMI data. As expected from the graphical summary, the effect of time was significant (*P*<.001), with the average estimated rate of change of .27 per month on the BMI scale. Controlling for age, individuals who completed more exercise Quests tended to have higher final BMI (*P*=.043); and individuals who played with less regularity also tended to have higher final BMI (*P*=.079).

**Table 3:** Results of multilevel modeling for changes in BMI.

Parameters	Estimate	SE	*P* value
Intercept	17.659	3.189	<.001
Time	-0.271	.036	<.001
Game Activities	.429	.195	.034
Gaming 1-2 hr/session	3.202	1.505	.040
TTM Physical Activity	-2.466	1.084	.029
Age	.223	.089	.017
SD (Intercept)	4.617		<.001
SD (Time)	.207		<.001
Residual	.235		
Rho	.001		

For nutrition knowledge, the average value at 30 days was 73.30 (± 13.59) compared to the mean at 90 days of 78.68 (± 12.66). This increase corresponds to Cohen’s d effect size of |.856| (large) based on the paired t-test. Results of the MLM are summarized in Table 4. Overall, knowledge increased with time (*P*<.001), with the estimated increase of 2.003 units per month in the study. Individuals who completed more exercise Quests had a marginally higher final level of nutrition knowledge (*P*=.089).

**Table 4:** Results of multilevel modeling for changes in nutrition knowledge.

Parameters	Estimate	SE	*P* value
Intercept	71.171	2.367	<.001
Time	1.980	.481	<.001
Food Quests	.926	.348	.011
Exercise Sparks	-.042	.018	.024
TTM^a^Physical Activity	12.656	3.426	<.001
SD (Intercept)	10.322		<.001
SD (Time)	2.324		<.001
Residual	3.022		
Rho	.001		

^a^Transtheoretical Model of Behavior Change for Physical Activity

## Discussion

Despite variable and limited adherence to the video game by the participants, our results demonstrate a relationship between SpaPlay play and changes in BMI and nutrition knowledge. This serves as preliminary evidence of the positive effects of incorporating Self-Determination and PENS theories into video game design to encourage behavior change towards healthy weight in women.

### Major Study Outcomes

A very promising result of the study is that the participants lost weight over the course of the three months. A significant and large change of almost one point on the BMI scale was observed, with the sample mean of 27 (SD 5.6) at baseline and 26.1 (SD 5.26) at the 3-month follow-up. The only other study that reported on BMI changes in the context of a game for diabetes management in adults [11] also found a drop in BMI, although of a smaller magnitude, .7 on the BMI scale over the course of 6 months. Sample demographics vary greatly between these 2 studies. At the same time, it is very encouraging to observe such a large effect. Further, individuals who completed more game activities (ie, cleaning the island, playing mini games), reported being in contemplation stage vs. preparation or action, were older, and who played 1-2 hours of video games per session tended to have higher final BMI scores.

According to the Transtheoretical Model [41-42], matching the intervention to cognitions and behaviors of individuals is of utmost important for ensuring success. Individualization of interventions for health behaviors, including diet and exercise, based on the individual’s readiness to change helps to move him or her along the behavior change trajectory. This theory found previous empirical support in traditional forms of interventions for dietary behaviors [43-45]. In the context of games, Lin et al. [14] carried out a qualitative study of participants engaged in step count linked to a virtual fish avatar. They found that those in the pre-contemplation or maintenance stages (both ends of the scale) were the least likely to change their daily steps. Findings from our study further support the relationship between behaviors and cognitions assessed through individuals’ readiness to change and their BMI. This has implications not only on how individuals are recruited but also clarifies the target audience and raises possibilities for individualizing game content explicitly depending on individual characteristics.

For nutrition knowledge, we also observed a large increase from the first month of assessment to follow up. While nutritional knowledge was intentionally not assessed at baseline, it is encouraging to see that the increase continued even after the first month of game play. Thus, it is likely that current findings are an underestimate of the game effect on nutritional knowledge. Similar findings of a large increase in knowledge of a food pyramid have been previously observed with a computer game to promote healthy diet in young adults (average age = 20 years) after 1 month of game play [10]. In our study, individuals who completed more food Quests and, interestingly, fewer exercise Sparks demonstrated higher levels of knowledge at post-test. In addition, women further along on the readiness to change their physical activity scale had significantly higher post-scores. These results support the fact that nutritional knowledge can be changed in a course of three months, even sporadic, game play intervention.

### SpaPlay Utilization

The overall low utilization of the SpaPlay program by the participants was a disappointing finding. On average, participants played for a week out of the 3 months, with only 50% playing more than 2 days. Gaps between logins were long, averaging 25 days (SD 14.23), and players largely exhibited an irregular play pattern as indicated by sporadic logins, estimated as an average standard deviation between logins of 16.48 (SD 7.66). Our previous usability studies [27,32] provided evidence for more frequent and consistent game play and generally good acceptance. Lower summaries from this study may be an artifact of design differences. The length of the qualitative study was 1 month, with interviews conducted regularly at one-week intervals, and interview questions informed by the play data [33]. This setup likely enforced more play than would occur naturally, without external reinforcement. These results may be a function of the instructions given to participants at baseline, where women were not explicitly told how often they should play the game. Rather, participants were expected to utilize the game as they would in real-life settings.

Further, we have examined game elements that were most frequently used. The participants favored briefer “Spark” activities over longer “Quests” that required a week-long commitment. It appears that short game features with immediate reinforcement were more appealing. A similar trend was found in our qualitative study and was largely expected.

We also found that some players engaged with the game more extensively than others, indicating a greater suitability of the game for some participants. This was supported by high variability scores in almost all game play elements, except for game and social activities. Women who, at baseline, reported being in the preparation and action stages of change on their diet played SpaPlay more frequently and consistently than those in the contemplation stage; they also completed more exercise Sparks. A theoretical application of the thranstheoretical model to game play adherence in the context of narrative-based games for health was proposed by Yin, Bickmore, and Montfort [46]; however, no empirical data were provided. Our findings present evidence that adherence to game play does depend on one’s cognitions and behaviors (as in preparation and action stages of change) and should be considered when designing and potentially individualizing games. Further research is needed to understand the construct of stages of changes more intimately in the context of games for health.

Another finding is that women who report playing video games 1-2 hours at a time had lower play frequency and consistency in the current game and completed fewer Sparks and Quests. Thus, a preference for spending a lot of time playing video games does not naturally translate into playing a health-related game. Taylor [47] discussed motivational factors for women who participate in multiplayer online gaming environments, which include social interaction, mastery, status, team participation, and exploration. The different style and context of SpaPlay seems to not appeal to this subgroup of women. This finding is important in understanding who might adopt or disregard this type of intervention. Interviewing women who engage in heavy bursts of game play could have shed more light into the nuances of the mismatch between their preferences and the current game, and could be a topic of a future investigation.

### Limitations

Results of the current study should be interpreted with caution in light of certain limitations. First, this study included a convenience sample of participants interested in playing computer games and who were at least contemplating losing weight. While this is a limitation, the sample is reflective of the population for whom we considered the game to be the most suitable and thus might resemble individuals who would seek a computer game to change their diet and exercise habits.

Second, the lack of a control group precludes complete certainty around the cause of weight loss and increase in nutritional knowledge. Given the pilot nature of the study, participants served as their own controls, which is strong but not absolute evidence of the game effectiveness. This could be an avenue of future research.

Third, more study follow-up data would have been beneficial for learning detailed lessons about players’ experiences. While our qualitative data from the previous study provides insight about satisfaction and acceptability [27,32], more data could provide additional insights into what works and what does not work when playing the game in more naturalistic settings. This should be considered in future studies, while considering participants’ fatigue from questionnaire responses.

Finally, all study measures were based on self-report. Since the current study is a part of an ongoing process of SpaPlay development, a new iteration of the game integrates objective measures of physical activity via Fitbit that is directly linked to game environment. Further investigations will make use of these objective data.

### Conclusion

As a concept, “games for health” is relatively new, with very few studies systematically and comprehensively evaluating validity and effectiveness. The current study incrementally contributes to the field, and highlights the complexity of several issues related to adherence, deployment in real life, and individualization. However, it is encouraging to see the ability of the game to change BMI and nutritional knowledge, both important targets of many health interventions.

## Abbreviations

BMI: body mass index
MLM: multilevel modeling
nlme: nonlinear mixed effects
PENS: Player Experience of Need Satisfaction
TTM: Transtheoretical Model of Behavior Change
